# Evaluation of patients with high burden of premature ventricular contractions by comprehensive transthoracic echocardiography

**DOI:** 10.1016/j.ijcha.2022.101124

**Published:** 2022-09-15

**Authors:** Raffaele Scorza, Kambiz Shahgaldi, Mårten Rosenqvist, Viveka Frykman

**Affiliations:** a Department of Cardiology, Danderyd University Hospital, Stockholm, Sweden; b Deparment of Cardiology and Clinical Physiology, Danderyd University Hospital, Stockholm, Sweden; c Karolinska Institutet, Department of Clinical Sciences, Cardiovascular Unit, Danderyd University Hospital, Stockholm, Sweden

**Keywords:** Premature, Ventricular, Contractions, Arrhythmia, Echocardiography, Examination

## Abstract

**Background:**

The prevalence and prognosis of premature ventricular contractions (PVCs) among individuals without structural heart disease are uncertain. Standard transthoracic echocardiography is a common method in evaluation of underlying cardiovascular disease and is recommended as a diagnostic method in PVC patients. However, it is unclear whether comprehensive echocardiographic examination can identify pathological findings in PVC patients with a normal standard echocardiogram.

**Method:**

We included forty consecutive patients with a high PVC burden (>10,000 PVCs/day) and normal findings at a standard echocardiogram and exercise test. All subjects were investigated by a comprehensive echocardiographic examination using parameters usually not included in a routine work-up. We compared the results with 22 age and sex-matched controls.

**Results:**

In six additional parameters–global longitudinal strain, right ventricular strain, septal-lateral delay, ventricular-arterial coupling, integrated backscatter and left atrial activation time–a statistically significant difference was shown between PVC patients and controls. Among these parameters, global longitudinal strain had a high reliability between operators.

**Conclusions:**

Despite normal findings at standard echocardiography, the PVC group showed signs of impaired heart function when more comprehensive echocardiography parameters were used.

## 1 Background

Premature ventricular contractions (PVCs) are common in clinical practice, both in patients with and without a history of structural heart disease [1], [2], [3], [4], [5], [6]. PVCs are known to be associated with a poor prognosis in persons with previous or current cardiac pathology, while their prognostic impact on subjects without known heart disease is not completely established [7], [8]. Transthoracic echocardiography (TTE) is recommended in the evaluation of PVC to exclude underlying structural heart disease [9]. Because of its availability and ability to show cardiac pathology, TTE is included in first-line evaluation of PVC patients [9]. However, it has been debated whether individuals with a high PVC burden and normal findings at echocardiogram are actually free of structural heart disease or if rather more comprehensive imaging methods should be used to identify subtle disease that PVCs could cause or ensue from [10]. Despite most PVC patients having a normal left ventricular (LV) Ejection Fraction (EF) at TTE, early signs of an eventual deterioration of the ventricular function may be missed by standard echocardiographic investigation [11], while comprehensive echocardiography could detect early electromechanical dysfunction [12], [13]. A prompt detection of ventricular *impairment is expected to be* clinically relevant, and echocardiography could be the most suitable non-invasive tool, being a widespread and inexpensive method of detection.

We aimed to investigate the additional role of comprehensive echocardiography in detection of subtle structural heart disease in patients with high PVC burden and a normal standard echocardiogram.

## 2 Methods

We included consecutive patients with at least 10,000 PVCs per day, and 22 healthy controls without cardiovascular disease. All patients had been evaluated at our Arrhythmia Outpatient Clinic by an experienced cardiologist and had undergone exercise test and standard echocardiography between January 2017 and December 2019. All patients had PVC burden established by Holter recording. Exclusion criteria were: previous history of ischemic heart disease, heart failure or other structural heart disease, pathologic result at standard echocardiography or exercise test. The controls were matched on group level for age and sex and were recruited on a voluntary basis.

Holter recording was initiated at the hospital and continued at home according to our clinical routine. The data were analysed manually by an experienced physician to ensure accuracy. A normal exercise test was defined as absence of exercise-induced depression of the ST-segment on ECG and absence of exercise-induced ventricular arrhythmia. All patients had a normal age-adjusted exercise capacity.

### 2.1 Echocardiography

All echocardiographic studies were performed with the patients in the left lateral decubitus position using a commercially available Vivid E9 machine (GE-Vingmed, Horten, Norway) equipped with a phased array transducer (M5S). A total of 3–5 cardiac cycles in sinus rhythm were recorded. All data were transferred to a dedicated workstation for offline analysis (EchoPAC PC version 204, GE-Vingmed, Horten, Norway).

Normal result at echocardiography was defined as LVEF > 55 % and right ventricular systolic function assessed by tricuspid annular plane systolic excursion (TAPSE) > 17 mm, absence of moderate to severe valve dysfunction, absence of regional wall motion abnormality, normal ventricular dimension, and normal wall thickness. The echocardiographic exams were reviewed by two additional independent and blinded examiners. In case of conflicting evaluations of the echocardiographic findings, the exam was considered normal if two of three examiners assessed the exam as such, and pathologic if two of the three examiners found signs of pathology.

Included PVC patients and controls were examined with comprehensive echocardiography, including functional parameters which are not routinely assessed in every echocardiography exam.

All the standard echocardiography parameters and Doppler measurements were performed according to the current recommendations [14], [15]. The examinations and analysis were performed by European Association of Cardiovascular Imaging (EACVI) certified sonographers.

LV Global longitudinal strain (GLS) by speckle tracking was calculated from four-, two- and three-chamber apical views. Tracing of the LV endocardium was performed manually, and the thickness of the region of interest (ROI) was adjusted to exclude the papillary muscles and the pericardium. The ROI was also adjusted to exclude the LV outflow tract and the left atrium. Reliable tracking of all myocardial segments throughout the cardiac cycle was confirmed visually. Views were excluded from analysis in case of insufficient tracking.

Mechanical dispersion was defined as the standard deviation of time to peak negative strain in 17 LV segments. RV GLS of the free wall and LA strain assessment was performed in concordance to the “How to perform right ventricular strain” paper [16] and “How to do LA strain” document [17]. LA stiffness, representing the change in pressure required to increase the volume of the atrium in a given measure [18], [19], was calculated as the ratio of E/é to LA reservoir strain. LV elastance as an index of myocardial contractility was calculated by modified single-beat method [20], employing systolic (SBP, mmHg) arm-cuff pressure to end-systolic LV volume.

Ventricular-arterial (VA) coupling was measured as ratio of end-systolic LV volume to Doppler-derived stroke volume [21]. Integrated backscatter (IBS) curves were acquired in the parasternal long-axis view in grey-scale 2D image, with framerates between 50 and 70 frames/s by locating a 5 × 5 mm sample volume in the basal-mid septum and inferolateral wall. A smaller fixed ROI (2 × 3 mm) was positioned in the pericardium in end-diastole as reference. Calibrated IBS was calculated by subtracting average pericardial IBS intensity from average myocardial IBS intensity of the septum and inferolateral wall and was expressed in decibels [22], [23]. The sample volume was tracked manually to maintain the same region throughout the cardiac cycle.

LA activation time was assessed by color-coded tissue Doppler in apical four-chamber view. A fixed ROI of 12 × 5 mm was placed on the lateral LA wall, just above the mitral annulus to acquire the tracing of mechanical activation in this area. The activation time was obtained by measuring the duration of the time delay between the onset of the P-wave on ECG and the peak of the Á -wave on the tissue Doppler tracing. LV diastolic function is assessed according to the latest recommendations from the American Society of Echocardiography and the European Association of Cardiovascular Imaging [15]*.*

### 2.2 Statistics

No power calculation was done before inclusion, as this is regarded as a descriptive pilot study. Calculation of mean values was done in Excel (Microsoft, Redmond, WA, USA). Categorical variables were compared using Chi-squared with Yates’ correction. A two-sided p-value of ≤ 0.05 was considered statistically significant*.* Continuous data were checked for normality with Shapiro-Wilk test. If normally distributed, they were compared with *t*-test; otherwise, they were compared with Mann-Whitney *U* test. Continuous data are presented as mean ± standard deviation or median and interquartile range (IQR) when appropriate. Nominal data are presented as number of cases (percent).

To determine interobserver variability of the parameters, six PVC patients and ten control subjects were measured by two different investigators, and interclass correlation coefficient for absolute agreement and coefficient of variation were calculated.

### 2.3 Ethics

The study was approved by the regional ethics committee (DNR 2014/670–31/4) and complied with the Declaration of Helsinki. All participants’ informed consent was obtained in written form. The study is registered at ClinicalTrials.gov with identifier NCT03370679.

## 3 Results

A total of 40 patients (18 female, 22 male) and 22 controls (6 female, 16 male) were included. Baseline clinical and echocardiographic characteristics are summarised in Tables 1 and 2. In the PVC-group the median number of PVCs/day was 18,000 with an interquartile range of 14,000–24,000. In a majority (35 of 40) of PVC patients, we also had access to PVC recording on 12-lead-ECG. The PVC morphologies from these patients are summarised in Table 3.

**Table 1:** **Clinical characteristics of PVC group and controls**. SD = standard deviation, IQR = inter-quartile range. Normally distributed continuous variables are presented as mean value and SD, non-normally distributed continuous variables are presented as median and IQR.

	**PVC-patients (n = 40)**	**Controls (n = 22)**	**p-value**
**Female, n (%)**	18 (45 %)	6 (27 %)	0.17
**Age, median (IQR)**	65 (45.5–74)	67.5 (61–72)	0.96
**Hypertension, n (%)**	11 (27.5 %)	0	<0.01
**Paroxysmal atrial fibrillation, n (%)**	5 (12.5 %)	0	<0.01
**Systolic blood pressure, mm Hg, mean ± SD**	136.54 ± 18.18	134.52 ± 19.49	0.70
**Diastolic blood pressure, mm Hg, median (IQR)**	80 (70–80)	75 (70–90)	0.97
**Body surface area, m^2^, mean ± SD**	1.9 ± 0.2	1.9 ± 0.2	0.37

**Table 2:** **Standard echocardiographic parameters in PVC-patients and controls.** SD = standard deviation, IQR = inter-quartile range. Normally distributed continuous variables are presented as mean value and SD, non-normally distributed continuous variables are presented as median and IQR.

**Echocardiographic parameter**	**PVC-patients (n = 40)**	**Controls (n = 22)**	**p-value**
**Left ventricular mass index, g/m^2^, mean ± SD**	74.4 ± 17.9	70.9 ± 16.2	0.43
**Stroke volume, mL, mean ± SD**	77.8 ± 18.1	83.2 ± 17.9	0.27
**Cardiac output, mL/min, mean ± SD**	4905 ± 1237	5421 ± 1195	0.11
**Left atrial volume index, mL/m^2^, mean ± SD**	32.4 ± 9.1	35.2 ± 7.8	0.20
**Left ventricular ejection fraction, %, mean ± SD**	58 ± 3.4	61 ± 4.2	0.01
**Tricuspid Annular Plane Systolic Excursion, mm, mean ± SD**	23.5 ± 3.1	22.9 ± 4.8	0.59
**Tricuspid Annulus max velocity, cm/s, mean ± SD**	12.8 ± 2.4	13.8 ± 2.5	0.08
**E-wave, cm/s, mean ± SD**	68.7 ± 15.9	76.7 ± 16.9	0.93
**E/A, median (IQR)**	1.1 (0.8–1.4)	1.17 (0.83–1.3)	0.01
**DT, ms, median (IQR)**	210 (190–250)	184 (150–210)	0.25
**é septal, cm/s, median (IQR)**	7.5 (6–10)	9 (6–10)	0.07
**é lateral, cm/s median (IQR)**	9 (8–12)	11 (9–13)	0.43
**E/é median, (IQR)**	7.5 (6–10)	8 (6–9)	0.72

**Table 3:** Distribution of 35 PVC-patients according to site of origin.

**PVC-Morphology**	**Number of patients**
Left ventricular	11
Right ventricular outflow tract	13
Right ventricular, other	7
Multifocal	4

### 3.1 Conventional echocardiography measurements

Eleven individuals in the PVC group had a diagnosis of hypertension, and five had paroxysmal atrial fibrillation. However, there was no significant difference in systolic and diastolic blood pressure measured before echocardiography. All participants had a normal systolic LV-function expressed as EF; although, controls had a significantly higher EF. Even E/A ratio was significantly higher in the control group, whereas the median value was above one in both groups.

### 3.2 Comprehensive echocardiography measurements

**Fig. 1:**
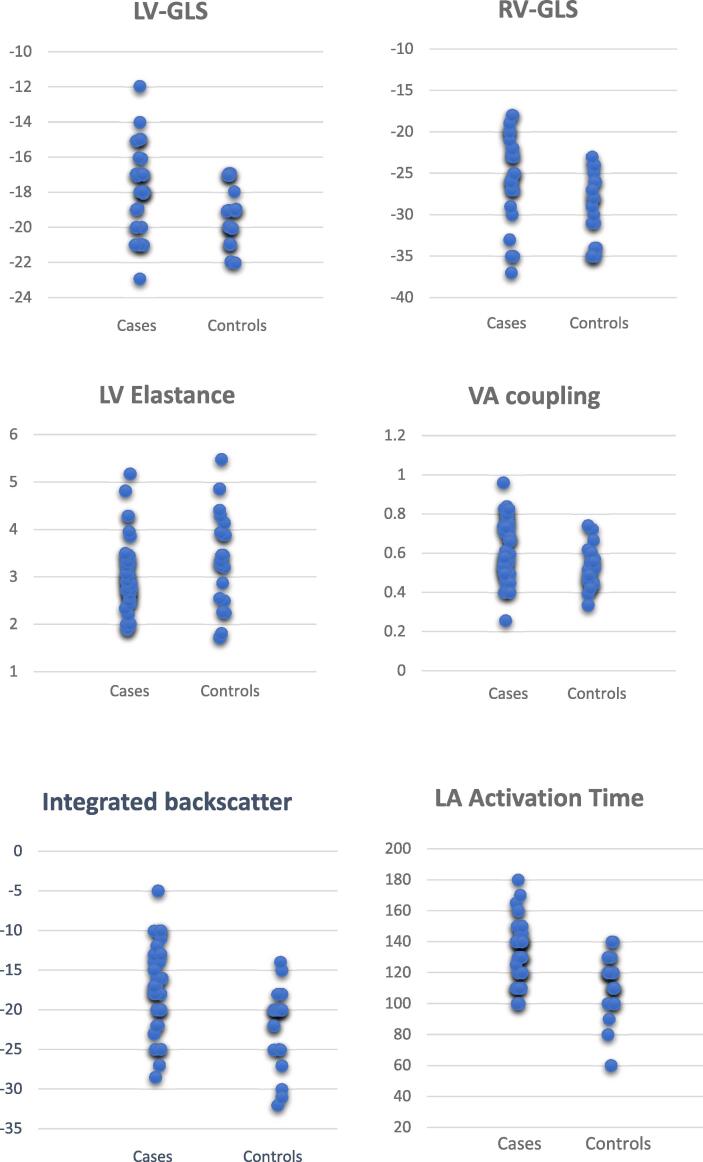
Jittered scatter plots of six parameters with statistically significant difference between cases and controls. LV = left ventricle, RV = right Ventricle, LA = left atrium. GLS = lobal longitudinal strain, VA = ventricular-atrial. GLS is measured in percent, elastance in mmHg/mL, integrated backscatter in dB, activation time in milliseconds and VA coupling is a ratio.

As illustrated in Table 4, the controls had a higher LV- and RV-strain performance compared to PVC patients. The controls also displayed a tendency towards higher LV stroke volume index, although this did not reach a statistical level. Jittered scatter plots for parameters showing a significant difference between the two groups are shown in Fig. 1.

**Table 4:** **Standard echocardiographic parameters in PVC patients and controls*.*** SD = standard deviation, IQR = inter-quartile range. Normally distributed continuous variables are presented as mean value and SD, non-normally distributed continuous variables are presented as median and IQR.

**Echocardiographic parameter**	**PVC-patients**	**Controls**	**p-value**
**Left Ventricular Global Longitudinal Strain, %, mean ± SD**	−17.76 ± 2.50	−19.4 ± 1.60	0.01
**Right Ventricular Global Longitudinal Strain, %, mean ± SD**	−25.26 ± 5.25	−28.58 ± 5.00	0.04
**Left Atrial Strain, %, mean ± SD**	−25.95 ± 8.74	−29.15 ± 5.00	0.17
**Left Atrial Stiffness (E/é /LAS) median (IQR)**	0.36 (0.195–0.45)	0.26 (0.215–0.35)	0.18
**Stroke Volume Index, mL/m^2^, median (IQR)**	38.64 (35.42 – 44.74)	45.4 (38–48.5)	0.05
**Left Ventricular Elastance, mmHg/mL, median (IQR)**	2.9 (2.60–3.3)	3.33 (2.55–3.94)	<0.01
**Ventricular-Arterial Coupling (EA/ELV), mean ± SD**	0.62 ± 0.15	0.52 ± 0.11	<0.01
**Integrated Backscatter, dB, mean ± SD**	17.2 ± 5.53	22.10 ± 4.87	<0.01
**Left Atrial Activation time, ms, mean ± SD**	133.95 ± 19.04	112.38 ± 19.47	<0.01
**Mechanical Dispersion, ms, median (IQR)**	48 (40–59)	45 (42–52.5)	0.5

The PVC patients exposed reduced LV elastance (2.9 vs 3.33 mmHg/mL, p < 0.01) and altered VA-coupling compared to the controls (0.62 ± 0.15 vs. 0.52 ± 0.11, p < 0.01).

Integrated Backscatter and LA activation time were also statistically different between the groups. Although, LA mechanical dispersion, LA strain and LA stiffness did not reach the level of significant difference between the groups.

Because the PVC group included patients with hypertension and atrial fibrillation we re-ran the analysis for the parameters showing a significant difference after exclusion of such patients. With the exception of LV elastance, all the parameters were confirmed to be significantly different between the two groups.

### 3.3 Feasibility and reproducibility

All measurements were not obtained in every patient due to insufficient image quality. LV GLS was obtainable in 29 cases (73 %) and 20 controls (91 %), RV GLS in 27 cases (68 %) and 19 controls (86 %), LA strain in 20 cases (50 %) and 20 controls (91 %), LA stiffness in 20 cases (50 %) and 20 controls (91 %), LV elastance in 39 cases (98 %) and 21 controls (95 %), VA coupling in 39 cases (98 %) and 21 controls (95 %), IBS in 37 cases (93 %) and 21 controls (95 %), LA activation time in 38 cases (95 %) and 21 controls (95 %), and mechanical dispersion in 29 cases (73 %) and 19 controls (91 %).

Re-measurements of six to ten randomly selected patients demonstrated a coefficient of variation between 12 and 20 % and inter-class correlation of 0.35 – 0.88 (95 % CI 0.54 ± 0.24), Table 5.

**Table 5:** **Comparison between measurements from two independent investigators in six PVC-patients and ten controls.** ICC = Interclass Correlation Coefficient, CV = Coefficient of Variation.

	**Investigator 1**	**Investigator 2**	**ICC**	**CV**
**Left ventricular Global Longitudinal Strain, %, median (IQR)**	−20 (−18.5- −20.75)	−19.75 -(18.25--21.5)	0.88	0.12
**Mechanical dispersion, ms, mean ± SD**	47.47 ± 8.06	42.53 ± 10.74	0.74	0.20
**Left Atrial Strain, %, mean ± SD**	−26.64 ± 7.66	−27.21 ± 8.02	0.63	0.16
**Lef Atrial activation time, ms, mean ± SD**	126.25 ± 19.19	108.13 ± 25.49	0.63	0.17
**Integrated Backscatter, dB, mean ± SD**	19.78 ± 4.35	17.88 ± 7.01	0.35	0.20

## 4 Discussion

In this descriptive pilot study, additional echocardiographic parameters that are commonly not included in routine examination were able to unmask possible signs of subtle myocardial pathology in patients with a high PVC burden and a normal echocardiogram. Evaluation of PVC patients with transthoracic echocardiography (TTE) is recommended in current guidelines and recently published reviews [24], [25], [26], [27]. Guidelines from American Heart Association/American College of Cardiology assign echocardiography in ventricular arrhythmia-evaluation a level 1B class of recommendation. A similar recommendation (1B) was given by European Society of Cardiology in 2015 and 2019 [28]. However, it has been debated whether conventional TTE is a sufficient instrument or if more advanced imaging methods should be used in some cases [10], [29]. Cardiac magnetic resonance (CMR) has been suggested as an alternative or completing examination, as a growing body of literature supports its use in PVC patients [29], [30], [31], [32]. However, CMR is an expensive and resource-consuming examination which is not as broadly available as TTE. The evidence for comprehensive echocardiography in PVC-evaluation is currently limited. Consequently, we identified additional echocardiographic measurements we estimated to be adequate to further analyse global LV contractility (Ventricular Atrial Coupling) and identify potential myocardial fibrosis (IBS, LA stiffness, LA activation time and mechanical dispersion) in PVC patients.

In our study all participants had normal LVEF and RV systolic function (by TAPSE) at standard TTE. When evaluated with GLS, PVC patients showed a significantly lower outcome both for left and right ventricle with a mean LV-GLS of −17.76 %. However, since this value is very close to the conventional cut-off value of −18 %, the clinical value of this difference is debatable and needs further exploration. Speckle tracking-derived GLS is a method for measuring the degree of deformation of the myocardium during the hemodynamic phases and has recently gained ground as a complement to LVEF [33], [34], [35], [36]. Speckle tracking echocardiography uses the movement of defined and recognizable areas within specific myocardial speckle patterns to analyse the deformation of the tissue. In 2010 Wijnmaalen and colleagues showed that this method could identify reduced left and right ventricular strain in PVC patients with normal standard TTE, compared to controls [10]. Radial, circumferential and longitudinal strain improved significantly after PVC-ablation but remained unchanged in untreated patients. Speckle tracking was even used by Lie et al. to assess left ventricular global longitudinal strain (GLS) and mechanical dispersion [37]. In this study the authors stated that PVC burden correlated with GLS and mechanical dispersion, but not with ejection fraction, the percent of PVCs being higher in patients with LV-dysfunction by GLS than in those with normal function. This finding could signify that speckle-tracking-measured GLS is able to identify subtle ventricular dysfunction. Among other studies using advanced echocardiographic techniques in PVC patients, we’d like to mention the one from Ling based on 40 subject with monomorphic frequent PVCs and 40 controls [38]. The results showed no significant differences in standard 2D-echocardiographical findings (including Ejection Fraction) between the two groups; however, parameters evaluated with 3D-speckle tracking echocardiography were significantly reduced in the PVC-group. In our study GLS had high reproducibility, as an ICC-value > 0.75 suggests excellent correlation between different measurements [39]. This could be an important finding given that there is evidence about GLS’ prognostic value for predicting adverse cardiovascular events [40]. GLS measurement was, however, not obtainable in all patients, which could limit its clinical implementation. A multivariate analysis could have been a good way to further assess the independent importance of this and other parameters, allowing for simultaneous observations in relation to each other. However, due to the missing values, an adequate multivariate analysis was difficult to carry out.

Functional evaluation in clinical practice has historically tended to focus on systolic ventricular function, as have published studies in the field. However, there is some evidence that PVCs affect diastolic function while systolic function is preserved [41], [42], [43]. An impaired diastolic function is often linked to rigid myocardial muscle, and tissue classification by integrated backscatter (IBS), a measure of the ultrasonic reflectance of the myocardium compared to the blood and the pericardium, has been used to detect myocardial fibrosis [44], [45], [46], [47], [48]. IBS PVC patients in our study had significantly lower IBS-values compared with controls; however, reproducibility was low, with an ICC value of 0.35. Among parameters investigating myocardial fibrosis, we obtained a higher ICC value for left atrial activation time, which has been used as a means to estimate electric conduction over the atria [49]. Atrial activation time was significantly longer in the PVC group. Since atrial fibrosis is known to be associated with atrial fibrillation [50], [51], [52], [53], and knowing that five PVC patients also had a diagnosis of paroxysmal atrial fibrillation, we re-ran the calculation after their exclusion. However, the difference between the two groups was still significant, pointing at a possible correlation between high PVC burden, fibrosis and risk for developing atrial fibrillation. LA strain and LA stiffness had lower feasibility in our study than in other publications, and we generally believe that this can be improved by software development [54], [55].

Ventricular end-systolic elastance is an echocardiographic parameter associated with both systolic and diastolic performance [56], [57] and can be described as the relation between pressure and volume inside the left ventricle. In our study elastance was significantly lower amongst PVC-patients, possibly summarising their combined systolic and diastolic impairment. Moreover, end-systolic elastance is a component in calculating Ventricular-Arterial coupling–which is also known to play an important role in global cardiac function [58], [59]–as the fundamental role of the cardiovascular system is to supply pressure and flow of blood to the tissues in different situations. Ventricular-Arterial coupling is measured as a ratio of arterial elastance and Left Ventricular end-systolic elastance. PVC patients had a significantly higher ratio in our study, indicating a worse ventricular performance. Although not significantly different from controls, the value for stroke volume index tended to be lower in PVC patients albeit within normal range[60].

A challenge within echocardiographic diagnostics is to define and secure reproducibility of the measurements, which depends on several factors, including quality of the examination, interpretation of the examiner and biologic variation [39]. As a result a relevant question is whether the same results can be obtained by the same investigator in other settings or by a different investigator in identical settings. However, even if completely identical settings cannot ultimately be achieved, due to biological variation, it is rational to focus on variation between investigators because the outcome from a single examination has a determining role in clinical practice. Amongst statistical methods to assess different aspects of reproducibility, we used inter-class correlation coefficient (ICC) and coefficient of variation (CV). ICC can be described as the measurement’s consistency in different settings (over time or between operators), while CV is a general measure of the data distribution, obtained by the ratio of standard deviation and mean value. Both measures can be presented as a percentage or a value between 0 and 1. A high ICC value and a low CV value indicates highly reliable data. In our study we computed ICC and CV for five parameters in 16 randomly chosen participants. Values for GLS suggest it to be highly reproducible, which is clinically relevant in these settings given that GLS was significantly lower in PVC patients, and there is growing evidence supporting its use. Even mechanical dispersion showed a good reproducibility, while left atrial strain, left atrial activation time, and integrated backscatter performed worse.

### 4.1 Limitations

No power calculation was done before the study, as it must be regarded as a pilot study. The echocardiographic examinations were carried out by experienced examiners. However, the image quality can be challenging for all measurement, and the calculations were based only on quality secured measurements. As a result some measurements were missing, which prevented us from performing an adequate multivariate analysis.

### 4.2 Conclusions

In this study individuals with a high PVC burden and a normal standard echocardiogram showed signs of cardiac dysfunction when evaluated with comprehensive echocardiography. The clinical significance of these findings must be assessed by larger longitudinal studies.

## 5 Sources of founding

This study was supported by grants from Stockholm Region, *Stiftelsen Hjärta* and the Swedish Heart-Lung Foundation (*Hjärt-Lungfonden*).

## Declaration of Competing Interest

The authors declare the following financial interests/personal relationships which may be considered as potential competing interests: RS and KS have no conflict of interest to declare. MR has received consultancy fees from Pfizer, Roche, Zenicor, Medtronic and Janssen. VF has received consultancy fees from Medtronic.
